# Use of simulation patients in the third section of the medical examination

**DOI:** 10.3205/zma001383

**Published:** 2020-12-03

**Authors:** Vivien Fritsche, A. F. Siol, Kai P. Schnabel, Daniel Bauer, J. Schubert, D. Stoevesandt

**Affiliations:** 1 Martin-Luther-Universität Halle-Wittenberg, Medizinische Fakultät, Dorothea Erxleben Lernzentrum, Halle (Saale), Germany; 2 Universität Bern, Medizinische Fakultät, Institut für Medizinische Lehre, Bern, Switzerland

## Abstract

In order to protect patients and students during the Covid 19 pandemic, the third section of the medical examination (M3) in Halle (Saale) was conducted in a modified form in accordance with the "Verordnung zur Abweichung von der Approbationsordnung für Ärzte bei einer epidemischen Lage von nationaler Tragweite” [1]. The one-day examination took place at the Dorothea Erxleben Learning Center (DELH) of the Martin Luther University Halle-Wittenberg on standardized simulation subjects. In contrast to previous years, all examiners were examined individually in internal medicine, surgery and their elective subject of the practical year. In the evaluations carried out, the standardized cases were assessed as consistent and fair by examiners and exam takers. Approximately 90% of the examiners could imagine to test a state examination with simulated patients again. After successful pilot testing, a study will be conducted in the coming exam to determine whether the substitution of real patients with simulated patients in the M3 exam can contribute to better standardization and objectivity while maintaining the same high level of acceptance in the exam. Whether the high acceptance will remain constant can only be checked in the course of the study.

## Zusammenfassung

Um Patient*innen und Student*innen während der Covid-19-Pandemie zu schützen, wurde der Dritte Abschnitt der Ärztlichen Prüfung (M3) in Halle (Saale) in abgewandelter Form gemäß „Verordnung zur Abweichung von der Approbationsordnung für Ärzte bei einer epidemischen Lage von nationaler Tragweite“ durchgeführt [1]. Die eintägige Prüfung fand im Dorothea Erxleben Lernzentrum (DELH) der Martin-Luther-Universität Halle-Wittenberg an standardisierten Simulationspersonen statt. Alle Prüfungsteilnehmer*innen wurden im Gegensatz zu den Vorjahren einzeln in Innerer Medizin, Chirurgie und ihrem Wahlfach des Praktischen Jahres geprüft. In den durchgeführten Evaluationen wurden die standardisierten Fälle von Prüfer*innen und Prüfungsteilnehmer*innen als in sich stimmig und fair bewertet. So konnten sich circa 90% der Prüfer*innen vorstellen, erneut ein Staatsexamen mit Simulationspersonen zu prüfen. Nach erfolgreicher Pilotierung soll im kommenden Examen im Rahmen einer Studie untersucht werden, ob die Substitution von Realpatienten durch Simulationspersonen in der M3-Prüfung zu einer besseren Standardisierung und Objektivität bei gleichbleibend hoher Akzeptanz im Examen beitragen kann. Ob die hohe Akzeptanz weiterhin konstant bleiben wird, kann nur im Verlauf der Studie überprüft werden.

## Introduction

The medical studies are completed with the third section of the medical examination (M3). The subjects Internal Medicine, Surgery and the respective elective from the Practical Year are each examined for 45-60 minutes over a course of 2 days. The examination group is normally composed of 4 exam takers and a four-member, usually medical, examination board. In the patient-based part of the examination the aim is to show that the candidate has mastered the techniques of taking a medical history, clinical examination methods and basic laboratory requirements and that the results can be evaluated. In addition, exam takers should be able to obtain and request the information necessary to make a diagnosis, recognize the different meanings and weightings of this information in making a diagnosis and critically evaluate this information in the context of differential diagnostic considerations [https://www.gesetze-im-internet.de/_appro_2002/BJNR240500002.html]. While in other countries such as Canada, the USA and Switzerland simulated patients (SP) have long been common in examinations [2], [3], [4], in Germany (in addition to the examination of real patients) this is only planned for the future [5]. 

## Project description

In order to protect real patients and to minimize physical interaction during the trial, the M3 trial was conducted in Halle (Saale) in May 2020 in a modified form according to the "Ordinance on Deviation from the Licensing Regulations for Physicians in the Event of an Epidemic Situation of National Significance" [1]. The one-day examination took place at the Dorothea Erxleben Learning Center (DELH). Thus, the SP established in teaching and the teaching infrastructure (rooms and computer technology) could be used in the examination situation.

In deviation from the norm, all exam takers were examined individually by 3 examiners in the fields of internal medicine, surgery and the respective elective subject. Thus the group size could be reduced from eight to four persons (the SP was not present during the group examination). A total of 64 exam takers examined on 17 days under the supervision of 59 examiners, each with a standardized, SP-based case. The cases were developed together with the responsible university lecturers based on real patient cases typical for the subject. Specific examination objectives were defined for each case (e.g., a subject must report a CT scan if he falls down stairs under Marcumar). The SPs were trained by means of written role descriptions and instruction by medical personnel on the day of the examination. Attention was paid to compliance with the applicable hygiene regulations. Physical examinations were only performed on phantoms (digital, rectal examination, cardiac auscultation, etc.). Examinations were not permitted due to the pandemic situation, but could be performed in the simulations outside the pandemic situation. Thus, a distance of 1.5 meters during the anamnesis and group examination was guaranteed. After the anamnesis discussion, for which 30 minutes were available, the participants had 10 minutes to fill out a request form (laboratory and further instrumental diagnostics). Independently of this, they received a simulated patient file tailored to the case, on the basis of which and taking into account the medical history, the patient report was prepared. In addition to the findings, the report should contain the diagnosis, prognosis, treatment plan and an epicrisis. In total, the subjects were given about 45 minutes to complete the report. The patient report and requirement forms were presented to the investigators before the one-hour interview.

**Figure 1:**
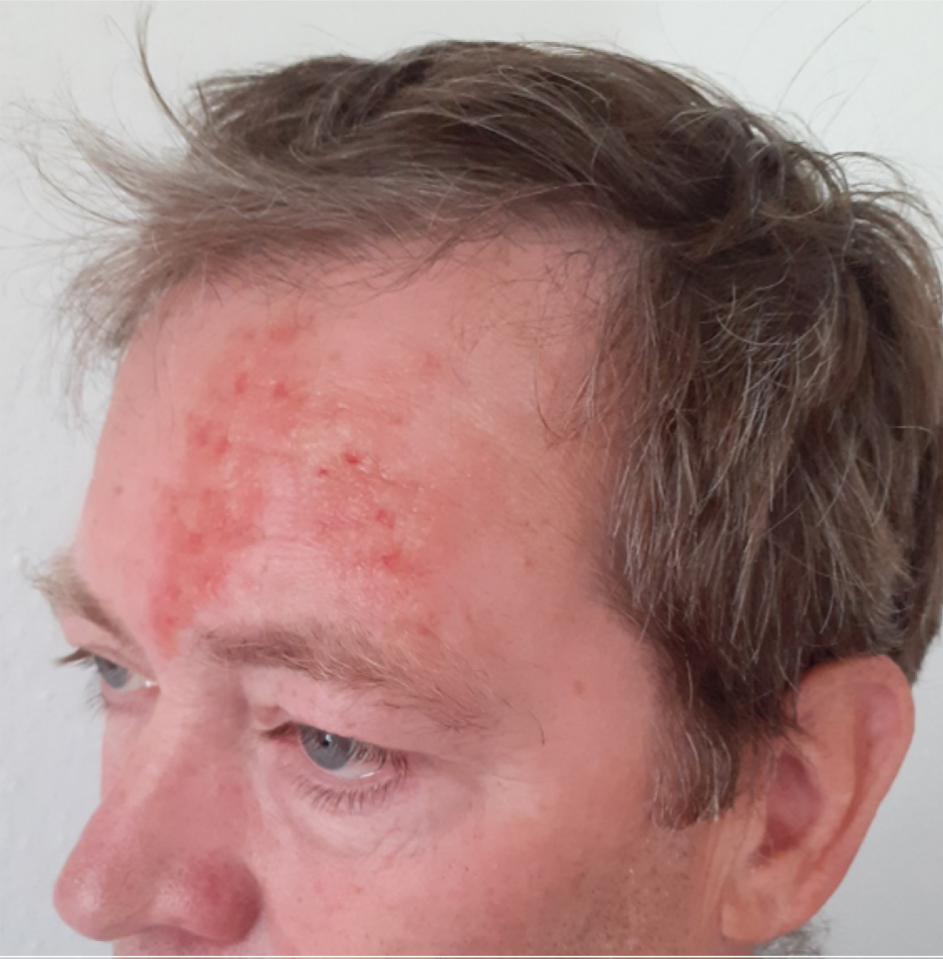
Simulation patient with Zoster ophthalmicus on the left side, implemented as manually colored 3D Probondo Transfer (Institute for Medical Education, Bern)

Where necessary for the understanding of the case or to narrow down differential diagnoses, the SPs were supported, e.g. by the application of disease-specific skin florescences or additional display of vital parameters on a monitor. Figure 1 (Fig. 1) shows an SP with a simulated zoster ophthalmicus. 

## Results

Using an evaluation questionnaire, we received feedback from 57 exam takers (89% response) and 38 examiners (64% response) in order to be able to make statements about the new examination format with SP and to make improvements in the course of the examination. 

**Figure 2:**
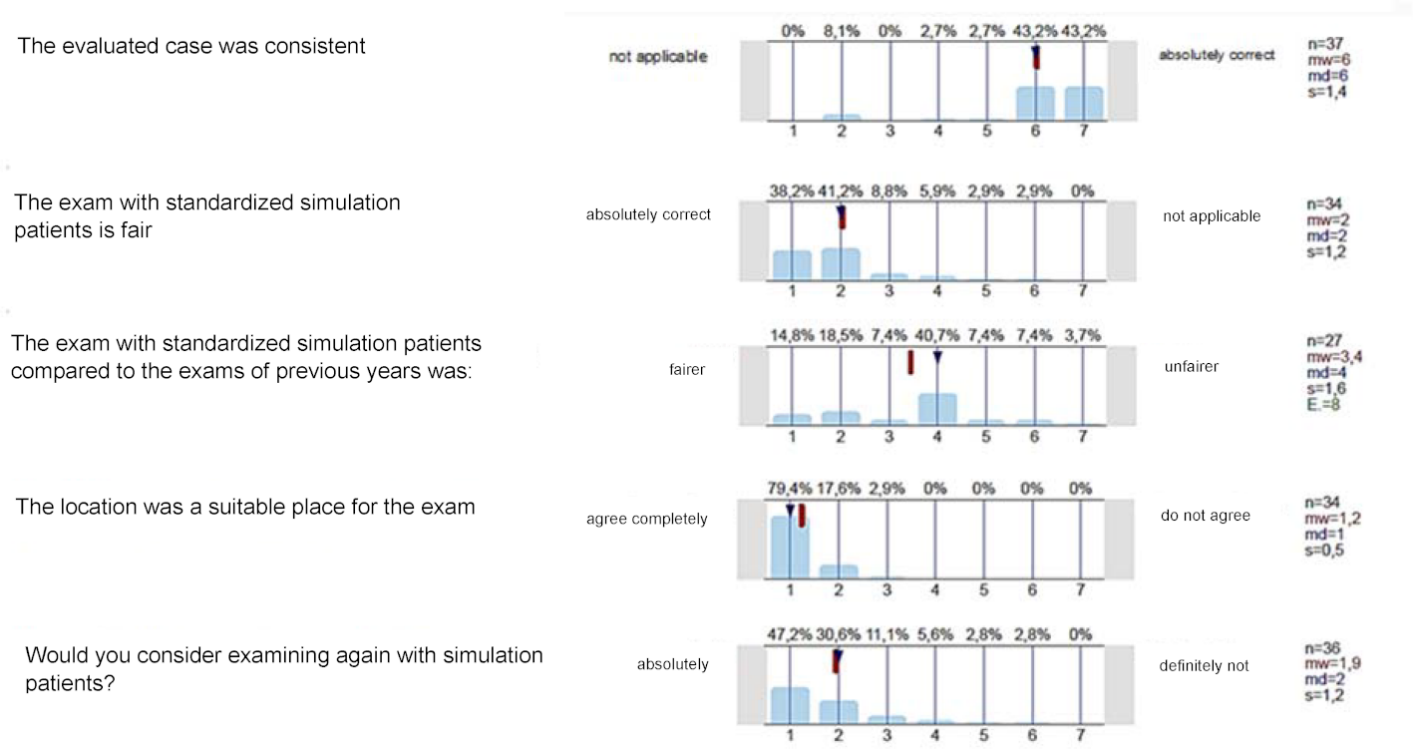
Evaluation data of examiners

**Figure 3:**
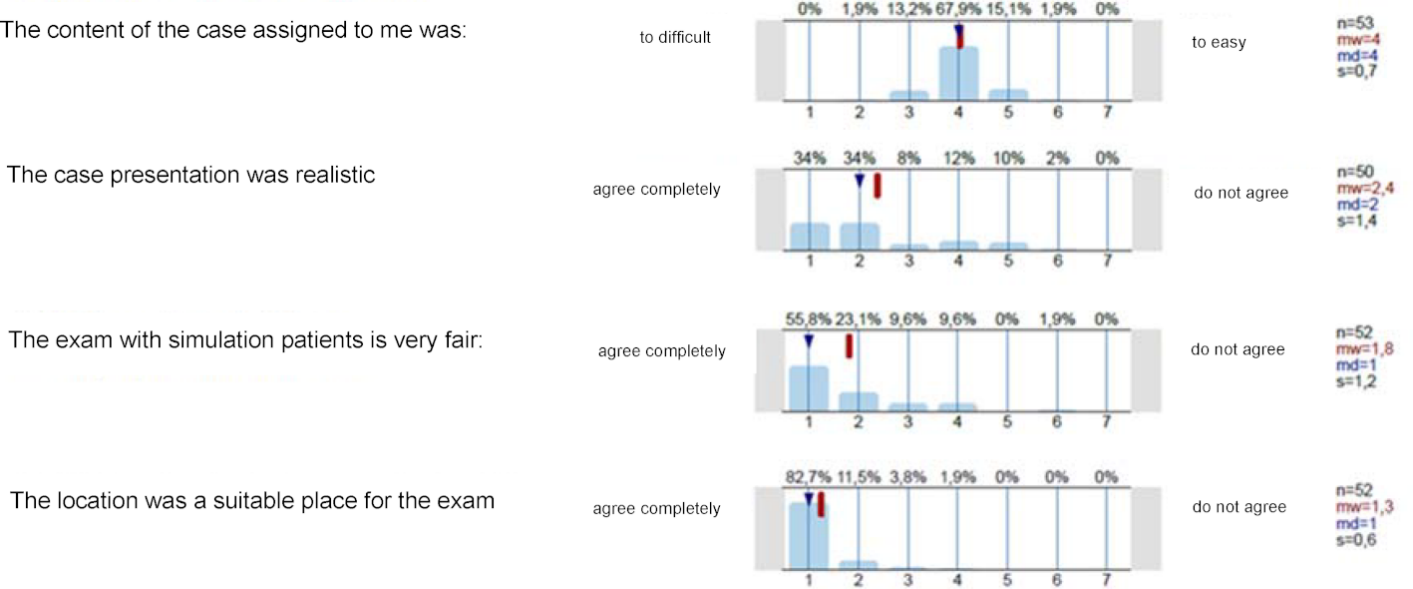
Evaluation data of exam takers

75% of the exam takers found the case presentation realistic. 86% of the examiners evaluated the cases as consistent. More than 75% of the examiners and exam takers rated the exam with standardized SP as fair. Compared to the classic M3 of previous years, the examiners rated the exam as equally fair or fairer in most cases. The overall oral exam grades did not differ compared to the previous year (mean value 2020: 1.9; mean value 2019: 2.0). When asked about the local conditions, almost all participants agreed that the learning clinic was a suitable place for the exam. Approximately 90% of the examiners can imagine taking a state exam again with SP. In free texts, some noted that this form of examination made it possible to achieve better comparability, because standardized cases were used and, thanks to the individual examination, they could ask similar questions in a group (see figure 2 (Fig. 2) and figure 3 (Fig. 3)).

## Discussion and conclusion

The use of SP is a nationally and internationally recognized component of medical education. Specially trained SPs take on the role of patients in order to facilitate exercise and examination scenarios and, in particular, to train communicative skills. A higher standardization of the exams could be achieved by reducing the number of patient cases, adjusting the severity of the exam in advance and the use of SP. Sommer et al. (2019) describe methodological advantages of SP, including the possibility to conduct reliable examinations [6]. Due to the Covid-19 pandemic, M3 was conducted with SP for the first time. Such tests are already established in Switzerland and other countries [2], [3], [4]. Due to the selected typical cases of disease, the concept could be transferred to other sites. With our evaluation data we could show that M3 testing at SP is basically suitable and is considered fair. We aim to continue M3 in the described manner in order to be able to consider the advantages and disadvantages of it in more detail on the basis of a larger study collective. Our data indicate that testing with standardized cases and SP as a possible testing format is perceived positively and should also be discussed in Germany as a routine for M3 testing.

## Competing interests

The authors declare that they have no competing interests. 

## References

[1] Bundesministerium für Gesundheit (2020). Verordnung zur Abweichung von der Approbationsordnung für Ärzte bei einer epidemischen Lage von nationaler Tragweite A. Problem und Ziel.

[2] Reznick R, Smee S, Rothman A, Chalmers A, Swanson D, Dufresne L, Lacombe G, Baumber J, Polde P, Lefasseur L (1992). An objective structured clinical examination for the licentiate: report of the pilot project of the Medical Council of Canada. Acad Med.

[3] Swygert K, Muller ES, Swanson DB, Scott CL (2009). The relationship between USMLE step 2 CS communication and interpersonal skills (CIS) ratings and the time spent by examinees interacting with standardized patients. Acad Med.

[4] Guttormsen S, Beyeler C, Bonvin R, Feller S, Schirlo C, Schnabel K, Schurter T, Berendonk C (2013). The new licencing examination for human medicine: from concept to implementation. Swiss Med Wkly.

[5] Jünger J (2017). Kompetenzorientiert prüfen im Staatsexamen Medizin. Bundesgesundheitsbl Gesundheitsforsch Gesundheitsschutz.

[6] Sommer M, Fritz AH, Thrien C, Kursch A, Peters T (2019). Simulated patients in medical education - a survey on the current status in Germany, Austria and Switzerland. GMS J Med Educ.

